# Long-term performance of a microbial electrolysis cell operated with periodic disconnection of power supply

**DOI:** 10.1039/c8ra01863d

**Published:** 2018-05-08

**Authors:** S. A. Hussain, M. Perrier, B. Tartakovsky

**Affiliations:** Département de Génie Chimique, École Polytechnique de Montréal C.P. 6079 Succ., Centre-Ville Montréal QC Canada H3C 3A7b; National Research Council of Canada 6100 Royalmount Ave., Montréal QC Canada H4P 2R2 Boris.Tartakovsky@cnrc-nrc.gc.ca +1-514-496-2664

## Abstract

This study describes a new approach for achieving stable long-term performance and maximizing the removal of chemical oxygen demand (COD) in a Microbial Electrolysis Cell (MEC). In the proposed approach, the MEC power supply is periodically disconnected, *e.g.* at a frequency of 0.1–0.5 Hz and a duty cycle of 90–95%. To evaluate the impact of such periodic power supply disconnection (on/off mode) on MEC performance, experiments were carried out in two flow-through MECs with activated granular carbon electrodes. The on/off operating strategy was applied to one MEC, while the other one was operated at a fixed voltage (control MEC). Long-term on/off operation resulted in progressive increase in COD removal efficiency (from 80% to 90%) and MEC current over time, while the control MEC showed stable but inferior performance. Furthermore, by changing the operating strategies and applying the on/off approach to the control MEC, its COD removal was increased from 78% to 83% and internal resistance decreased. The proposed on/off mode of operation can be used to develop a high-rate MEC-based wastewater treatment system.

## Abstract 2

This study describes a new approach for achieving stable long-term performance and maximizing removal of chemical oxygen demand (COD) in a Microbial Electrolysis Cell (MEC) by periodic disconnection of the MEC power supply.

## Graphical abstract

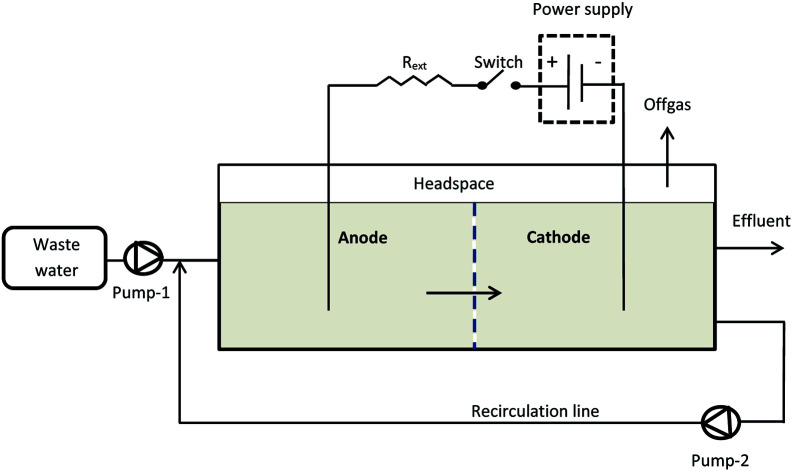

## 1. Introduction

Biodegradation of organic materials in a microbial electrolysis cell (MEC) can be used to develop a net energy-positive wastewater treatment process. Indeed, hydrogen production from wastewater in a MEC was one of the first proposed MEC applications.^1,2^ Methane production is often observed at the MEC cathode, either due to hydrogen conversion to methane by hydrogenotrophic methanogenic microorganisms, or by direct electromethanogenesis.^3–6^ A recently proposed MEC-based wastewater treatment technology takes advantage of the fast conversion of hydrogen and carbon dioxide to methane by utilizing a flow-through membraneless MEC design, which combines electricigenic (anodophilic) and conventional anaerobic pathways of COD degradation.^7^ This technology is based on bioelectrodes made of granular activated carbon, which require relatively long startup times to develop an electrochemically active microbial biofilms at the anode and cathode. A strategy capable of accelerating this process is required for practical application of this MEC configuration.

Recently, several optimization approaches were proposed to enable real-time optimization of Microbial Fuel Cells (MFCs). In particular, increased power production in a MFC operated with periodic disconnection of external resistance or using pulse width modulated (PWM) resistance connection was demonstrated.^8,9^ Furthermore, a simple equivalent electrical circuit (EEC) model of an MFC and a parameter estimation procedure adapted to the PWM mode of operation was developed and successfully used to achieve real time monitoring of MFC performance.^10^

So far, very few attempts were made to apply similar approaches to MEC optimization and/or monitoring. In one study, the production of hydrogen gas in a MEC was maximized by real-time optimization of applied voltage.^11^ Here, the applied voltage was modified in real time in order to minimize MEC apparent resistance by using a Perturbation-and-Observation (P/O) algorithm. In a more recent study, an equivalent electrical circuit model of MEC was developed and used for real-time monitoring of internal resistance and capacitance,^12^ which requires intermittent disconnection from power supply. This approach was shown to be capable of successfully tracking changes in the operating conditions, including variations in the influent chemical oxygen demand (COD) concentration.

Constantly changing wastewater composition and COD concentration combined with a requirement of the treated effluent COD concentration to be below a certain regulatory threshold provides a limited number of controllable inputs in a wastewater treatment process. Since growth and metabolic activity of electroactive bacteria are directly proportional to the applied voltage, it could be expected to have significant impact on MEC performance.

This study attempts to adapt the approach of MFC operation with intermittent (on/off) connection to an electrical load, which was shown to greatly improve MFC performance^8^ to operation of an MEC. Accordingly, the impact of MEC operation with intermittent disconnection of power supply on current and COD removal is investigated. Experiments are carried out in two MECs with flow-through porous bioelectrodes made of granular activated carbon.^7^

## 2. Material and methods

### 2.1 Feed composition and analytical methods

The stock solution of feed was either acetate-based (synthetic wastewater) or composed of brewery wastewater. Acetate stock solution was composed of (per L) anhydrous sodium acetate (40 g), yeast extract (0.83 g), ammonium chloride (18.7 g), potassium chloride (74.1 g), potassium phosphate dibasic (32.0 g) and potassium phosphate monobasic (20.4 g). The stock solution was diluted with deionized water to obtain the desired acetate concentration. Also, solution of trace metals was added to synthetic wastewater (1 mL per L) to provide essential microelements. The composition of the trace metal solution can be found elsewhere.^4^ The resulting synthetic wastewater solution had a conductivity of 15–17 mS cm^−1^. Brewery wastewater with an average total COD concentration of 6.7 g L^−1^ was obtained from Fleischmann's Yeast Ltd (Calgary, AB, Canada). It too was diluted with deionized water to obtain the desired influent COD concentration of 2000 mg L^−1^. This solution had a conductivity of 5–7 mS cm^−1^.

Biogas production was measured with the MilliGascounter (Ritter Apparatus, Bochum, Germany). Biogas composition was evaluated using an HP 6890 gas chromatograph (Hewlett Packard, Palo Alto, CA, USA). Acetate concentration was analyzed with a second Agilent 6890 gas chromatograph (Wilmington, DE, USA) equipped with a flame ionization detector. Method details are provided elsewhere.^13^

### 2.2 MEC design and operation

**Fig. 1:**
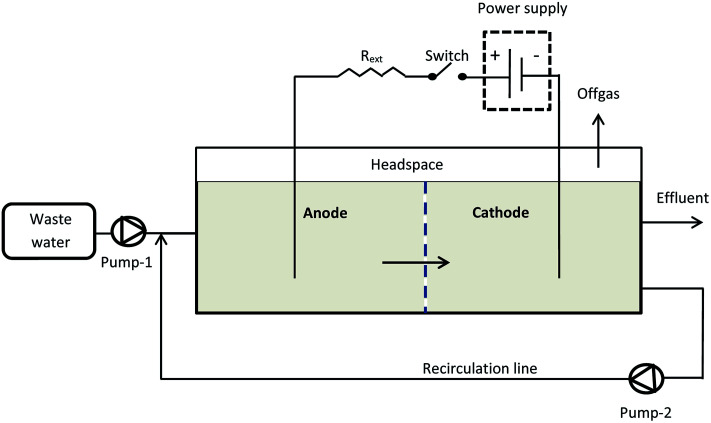
Experimental setup.

Two horizontal flow rectangular MECs (MEC-A and MEC-B) were constructed using Plexiglas plates. Each MEC had equally sized anode and cathode compartments separated by a non-conductive porous material (geotextile cloth). Granular activated carbon (GAC) was used as both the anode and cathode material occupying the entire volume of each electrode compartment. Each MEC had a total liquid volume of 1.7 L, with a headspace of 0.3 L and was equipped with off-gas exit lines, which collected biogas from both electrode compartments. An external recirculation line was used to ensure adequate mixing. The recirculation rate was 9.6 mL min^−1^. The MECs were operated as flow-through reactors, where the influent stream entered the anode compartment and the effluent was collected at the end of the cathode compartment as shown in Fig. 1.

Each electrode compartment was inoculated with 20 mL of homogenized anaerobic sludge (Lassonde Inc., Rougemont, QC, Canada), which had a volatile suspended solids content of 22–25 g L^−1^. The MECs were operated at room temperature (22–24 °C). Initially the MECs were fed with synthetic (acetate-based) wastewater at a flow rate of 2 L d^−1^, *i.e.* a hydraulic retention time (HRT) of 0.85 day was maintained, as calculated based on the total reactor volume. The stock acetate solution and the dilution water were fed using peristaltic pumps. Experiments were carried out by feeding the MECs at an influent acetate concentration of 1000 mg L (1070 mg L^−1^ as COD). Following MEC operation on synthetic wastewater the influent stream was changed to brewery wastewater.

Methane yield (L g^−1^) was calculated according to the following equation:1
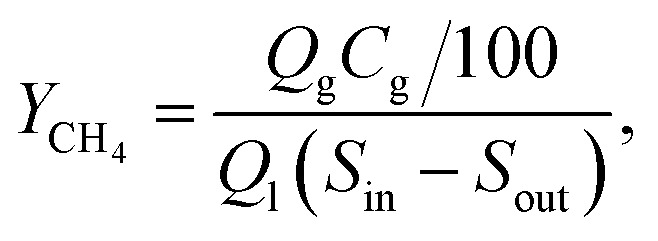
where *Q*_g_ is the biogas flow rate (L d^−1^), *C*_g_ is the percentage of methane in biogas (%), *Q*_l_ is the liquid flow to and from MEC (L d^−1^), *S*_in_ and *S*_out_ are the influent and effluent acetate concentrations (g L^−1^ as COD).

Coulombic efficiency (CE) was calculated as the ratio between the total Coulombs transferred to the anode from the carbon source (estimated based on MEC current measurements) and the theoretical maximum estimated based on COD (acetate) consumption.^14^

### 2.3 Electrical measurements and electrochemical techniques

The MEC was operated at an applied voltage of 1.5 V using a PW18-1.8AQ power supply (Kenwood Corp, Tokyo, Japan) interfaced with a computer. Current was measured with a 15 Ohm shunt resistance (*R*_ext_) according to the diagram shown in Fig. 1. MEC operation with periodic power supply disconnection (on/off operation) was achieved using a relay controlled by LabJack U3-LV (LabJack Corp., Lakewood, CO, USA) data acquisition board, which was also used to record power supply voltage (*U*_s_), and voltage measured across the MEC (*U*_MEC_). Voltage across the external resistor (*U*_R_) was calculated as the difference of *U*_MEC_ and *U*_S_ values and then used to calculate MEC current.

Software for on/off MEC operation and data acquisition was written in Matlab R2010a (Mathworks, Natick, MA, USA). After certain (user specified) time, the computer program calculated and recorded the average current (*I*_avg_) and power (*P*_avg_) per each on/off cycle. These values were calculated as follows:2
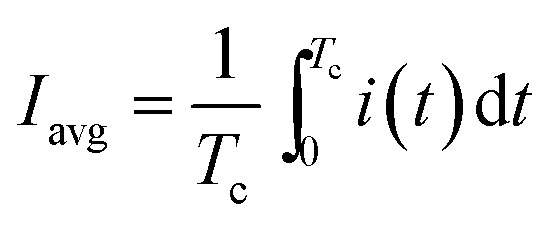
3
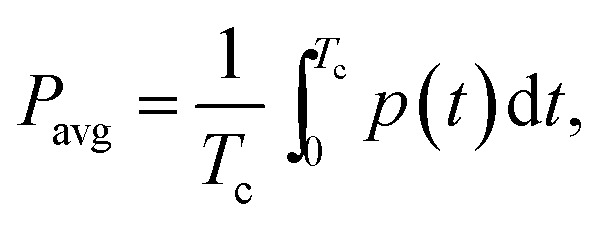
where *T*_c_ is the cycle length, *i* and *p* are the current and power at time *t*. Here, *p* is calculated as

4 *p*(*t*) = *i*(*t*)*U*_MEC_

Total internal resistance of the MECs was estimated using a voltage scan technique (Escapa *et al.* 2012). Voltage scans were performed by changing the applied voltage between 0.2 and 1.6 V with 10 min intervals after each voltage change to allow the outputs to stabilize. The resulting curves were used to estimate total internal resistance as a slope of the linear part of the voltage *vs.* current plot.

## 3. Results and discussion

MEC operation with intermittent disconnection of power supply was studied in membraneless horizontal flow MECs.^7^ It might be mentioned that the concept of a flow-through MEC is somewhat different from the original MEC design intended for hydrogen production at the cathode separated from the anode by a proton exchange membrane.^1^ The flow-through design facilitates exchange of ions and CO_2_ between the electrode compartments. The presence of CO_2_ at the cathode results in H_2_ conversion to CH_4_ by hydrogenotrophic methanogenic and electroactive microorganisms.^4^ Overall, the flow-through MEC design combines methanogenic and electrogenic microbial activities, resulting in an increased COD removal rate and improved methane yield, as recently demonstrated by Tartakovsky *et al.*^7^

Operation of MEC-A and MEC-B on synthetic wastewater consisted of four distinctive phases. Phase 1 (start-up) lasted 10 days and was followed by 3 phases of operation under differing conditions for either MEC. The startup phase for both MEC-A and MEC-B was initiated with a fixed applied voltage of 1.5 V and a continuous influent acetate concentration of 1070 mg L^−1^ as COD. Once stable current was observed, phase 2 was initiated by subjecting MEC-A to a periodic connection/disconnection of the power supply (on/off operation), while MEC-B continued to be maintained at a fixed applied voltage.

**Fig. 2:**
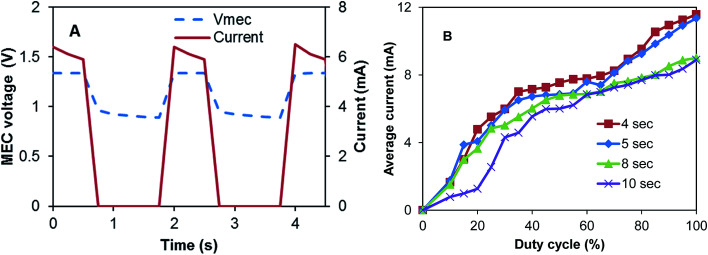
(A) MEC-A voltage and current observed at *D* = 12.5% and *T*_c_ = 2 s, and (B) average (per cycle) current calculated at several *D* and *T*_c_ values.

Fig. 2A illustrates the approach of MEC-A operation in the on/off mode. As can be seen from this graph, the highest current is observed immediately after the power supply is connected to the MEC electrodes. During the “on” part of the cycle (constant applied voltage), the current somewhat decreases and then drops to zero once the power supply is disconnected. Interestingly, after each disconnection of the power supply MEC voltage only drops to about 0.9 V and then only slightly decreases during the off part of the cycle. Such voltage dynamics could be attributed to the significant internal capacitance of the electroactive biofilm^15–17^ and large surface area of GAC electrodes. Also the existence of an internal electromotive force in this flow-through membraneless bioelectrochemical system can be hypothesized, as discussed in our previous study.^12^

To observe the impact of frequency with which power supply is connected to the MEC (duty cycle, *D*) and the length of the connection/disconnection cycle (*T*_c_) on average current, MEC-A was operated at several combinations of *T*_c_ and *D*. In particular, *T*_c_ was varied between 2 and 10 s and for each *T*_c_ value *D* values were increased in 5% increments from 10% to 100%. Fig. 2B shows the resulting dependencies of average current calculated according to eqn (2) on *T*_c_ and *D* values. At all values of *T*_c_ a near linear increase of current was observed with increasing *D* (between *D* = 10–40%). A fast increase of average current was followed by a slower increase at *D* values between 40–70% (especially at *T*_c_ values of 4 and 5 s) and then a somewhat faster increase at *D* values above 70%. As a result, at all tested *T*_c_ values the highest average current was observed at *D* = 100%, *i.e.* the highest current corresponded to a fixed applied voltage. This result is contrary to the observation of a higher power production and current in an MFC operated with periodic or pulse-width modulated disconnection of external resistance.^8,9^ In this case the increased power production could be attributed to charge storage in the anodic biofilm and reduced activation losses.

**Fig. 3:**
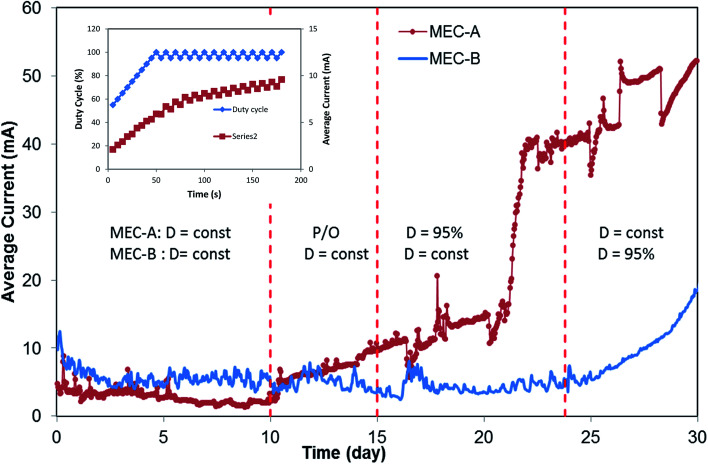
MEC-A and MEC-B average current observed during periodic disconnection and fixed applied voltage modes of operation. MEC-A on/off operation was started on day 10. MEC-B on/off operation was started on day 24, as indicated by vertical dashed lines. Inset shows results of the P/O test performed on day 12 to optimize *D*.

A second attempt at *D* optimization involved a Perturbation-and-Observation (P/O) algorithm, which was used to impose step-wise 5% changes of *D* starting from an initial *D* value of 50%. A detailed description of the algorithm can be found elsewhere.^18^ Results of this test are shown in Fig. 3 (inset). This test was carried out at a *T*_c_ = 5 s with each *D* value maintained for 30 min. Once again, the highest average current was obtained at *D* = 100% (fixed applied voltage). Interestingly, although the test did not indicate the existence of optimal duty cycle, which would correspond to the highest average current, progressive increase of the current towards the end of the test was noted. The P/O algorithm was designed to decrease *D* back to 95% if *D* = 100% is reached. Accordingly, *D* fluctuated between 95% and 100% for a significant part of the test, as can be seen from the graph (Fig. 3 inset). The P/O optimization test was repeated over a period of several days. Overall, the frequency and duty cycle tests continued for 5 days (between days 10–15). Although each test indicated that the highest average current corresponds to fixed applied voltage, a pronounced increase in the current of MEC-A was noticed by day 15. At the same time, the current of MEC-B operated at a fixed applied voltage remained unchanged, as can be seen from Fig. 3.

As a result of this observation, in phase 3 of the test MEC-A continued to operate in the on/off mode at a *D* of 95% and *T*_c_ of 5 s, while maintaining MEC-B at *D* = 100% (fixed applied voltage). The resulting long-term performance of the two MECs in terms of average currents is shown in Fig. 3 (days 15–24). Clearly, MEC-A operation in the on/off mode led to a progressively increased average current. At the same time, MEC-B current did not improve, although it was operated at the same applied voltage, flow rate and influent carbon source concentration as MEC-A. While some differences between performances of the two MECs might be expected due to the biological nature of this bioelectrochemical system (*e.g.* variations in biofilm growth rates and/or microbial populations, inhomogeneity of flow through granular carbon electrodes, *etc.*), the observed differences in average currents by the end of the test on day 24 were remarkable. While MEC-A current exceeded 40 mA, MEC-B current remained at around 6 mA, same as on day 10.

Interestingly, a sharp increase in MEC-A average current between days 19–23 was noticeable. It can be hypothesized that this increase reflected the proliferation of electroactive microorganisms at the cathode. Slow growth of bioelectrochemically active cathodic biofilms during MEC operation with biocathodes was previously observed.^3,19–21^ Accordingly, we can attribute current increase between days 10–19 to the anodophilic biofilm formation at the anode and the increase between days 19–23 to the proliferation of electroactive microbial populations at the cathode, as well as further biofilm growth at the anode.

**Fig. 4:**
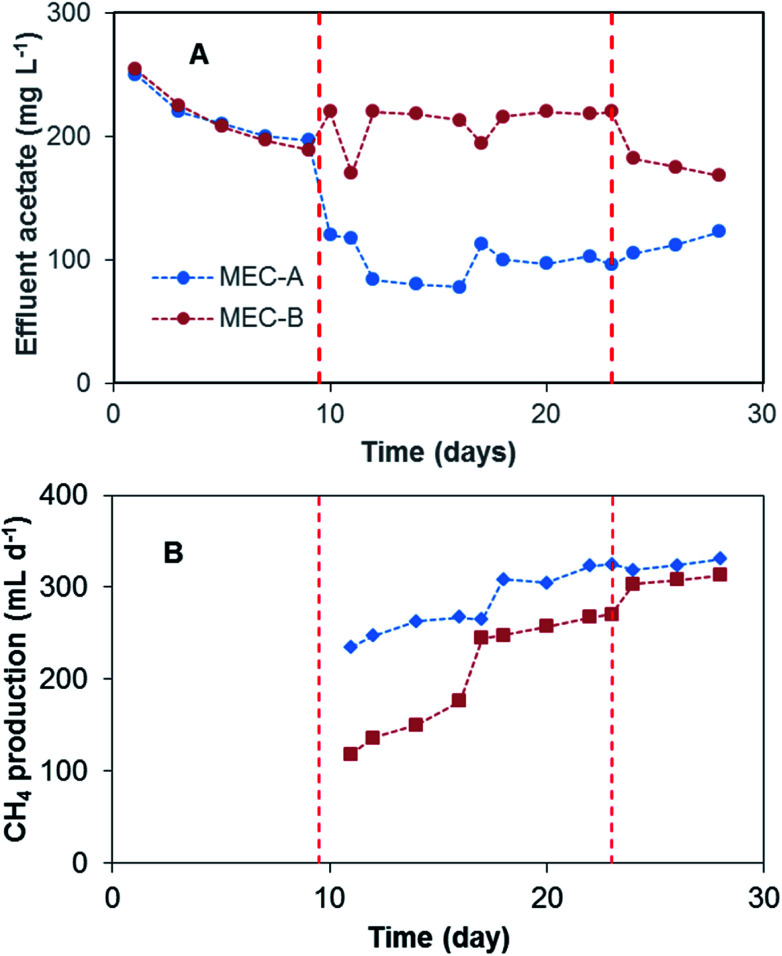
(A) Effluent acetate concentration and (B) CH_4_ production in MEC-A and MEC-B reactors. MEC-A operated in on/off mode between day 10–23 and MEC-B between day 24–30. CH_4_ measurements were started on day 11 of the test.

Additional confirmation of better MEC-A performance was obtained from the analysis of acetate concentration in the effluent and its comparison with the corresponding effluent values in MEC-B. As shown in Fig. 4, shortly after the startup of the on/off operation the effluent acetate concentration of MEC-A decreased considerably, while it remained at the same level in the MEC-B effluent. It can be concluded that MEC-A provided better COD removal efficiency (90% and 78% for MEC-A and MEC-B, respectively). Also, the COD removal efficiency of MEC-A increased from 80% (day 9) to 90% (day 23). Fast current increase in MEC-A between days 19–23 was not accompanied by a corresponding decrease in the effluent acetate concentration (Fig. 4). It can be argued that at already low acetate concentration of 103–117 mg L^−1^ corresponding to this period of operation, the kinetics of COD consumption was carbon source – limited for methanogenic microorganisms. Anodophilic microorganisms are known to exhibit a higher affinity for acetate,^22,23^ thus the increase in average MEC-A current was indicative of the population shift, where a larger part of acetate was consumed by the electroactive microorganisms due to increased current.

The contribution of electroactive microorganisms to the overall COD removal can be estimated based on coulombic efficiency (CE) calculations.^14^ These calculations suggest that during the first 10 days of MEC-A and MEC-B operation the electroactive microorganisms only contributed 4–5% to the overall COD removal. MEC-A operation with intermittent disconnection of the power supply resulted in nearly an 8 fold current increase, which can be attributed to the proliferation of the electroactive microorganisms at both electrodes. Accordingly, current and COD measurements between days 20–23 yielded CE values of 20–23% for MEC-A, while CE estimations for MEC-B remained at 4–5%. Importantly, current increase during the on/off mode of MEC operation served as a confirmation of the bioelectrochemical activity. Throughout the tests both MECs were operated at an applied voltage of 1.5 V, which is higher than 0.6–1.2 V often used for MEC operation.^24^ Ohmic losses and significant overpotentials of GAC electrodes increased the threshold for water electrolysis to approximately 1.8 V.^25^ Indeed, voltage measurements between two titanium wires placed approximately at a distance of 1 cm from current distributors in each electrode compartment showed a voltage of 1.0–1.2 V thus confirming significant ohmic losses in GAC electrodes.

To confirm the hypothesis of improved MEC performance due to periodic power supply disconnection, in phase 4 of the test the operation strategies of the two MECs were changed. On day 23 MEC-B was connected to the electronic switch to enable the on/off operation (*D* = 95%), while MEC-A was connected to fixed applied voltage. This mode of operation continued until day 30. Shortly after the startup of MEC-B on/off operation the average current improved, reaching 22 mA by the end of this phase of the test (Fig. 3). At the same time, MEC-A current remained nearly constant at 40–43 mA. Although MEC-B current did not reach the level observed in MEC-A, the increase of the average current was accompanied by a decrease of the effluent acetate concentration (Fig. 4) and the COD removal efficiency increased from 78% to 83%. Overall, the test strongly suggested a positive link between the improved MEC performance and the on/off mode of operation, at least at a *D* value of 95%.

**Fig. 5:**
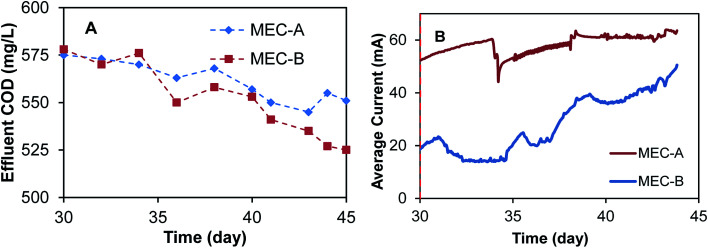
MEC-A and MEC-B current and effluent COD concentrations during operation on brewery wastewater. MEC-B was operated in the on/off mode, while MEC-A was operated at a fixed applied voltage.

Following MEC-A and MEC-B operation on synthetic wastewater, the feed solution was changed on day 30 to brewery wastewater with a COD content of approximately 2000 mg L^−1^. MEC operation on brewery wastewater was continued for 15 days. With MEC-B operating in the on/off mode, and MEC-A operating at a fixed applied voltage of 1.5 V. Fig. 5 shows the observed effluent COD concentrations and currents. As expected, a change in the influent COD concentration from 1060 mg L^−1^ to 2000 mg L^−1^ led to a sharp increase in the effluent CODs with similar values observed in both MECs. Over the course of the 15 day test the effluent CODs declined in both MECs, apparently due to an adaptation response of microbial populations to the new feed type and higher organic load. However, the effluent COD concentration of MEC-B decreased faster and was lower than in MEC-A by the end of the 15 day test, although the difference was relatively small (Fig. 5A). This improvement in MEC-B performance coincided with the progressive increase in current, as shown in Fig. 5B. It should be emphasized that MEC-A current remained unchanged.

On day 20 of MEC operation and at the end of the test (day 45) internal resistances of both MECs were estimated by carrying out voltage scan tests. The estimated total internal resistance values obtained in these tests are given in Table 1. Clearly, the on/off mode of operation helped to reduce *R*_int_ of MEC-A during its operation on acetate. Also, *R*_int_ of MEC-B was substantially decreased by the end of the test, as compared to its initial value.

**Table 1:** Internal resistance estimations for MEC-A and MEC-B operated on synthetic (acetate) and brewery wastewaters. Total internal resistance estimations are based on voltage scan tests

Wastewater	*R*_int_ (Ohm)	
	MEC-A	MEC-B
Acetate (day 20)	35.5	270.3
Brewery (day 45)	5.5	5.2

The observation of improved long-term performance in a MEC operated with periodic disconnection of applied voltage can be attributed to multiple factors, including process electrochemistry and microbiology. Indeed, improved performance of an MFC operated with periodic disconnection from electrical load was hypothesized to be related to reduced activation losses,^8^ a hypothesis, which requires a thorough electrochemical investigation. Changes in microbial populations due to complex non-linear dynamics of electroactive and non-electroactive microbial populations subjected to periodic voltage variations could be another plausible explanation. Periodic disconnection of applied voltage might affect dynamics of carbon source consumption by electroactive (anodophilic) microorganisms at the anode and therefore carbon source distribution in the biofilm. Considering the coexistence of anodophilic and methanogenic populations in the biofilm,^23,26^ the methanogenic populations might also be affected. Furthermore, periodic variations in the anodophilic activity might also affect electroactive microbial populations at the cathode.

**Fig. 6:**
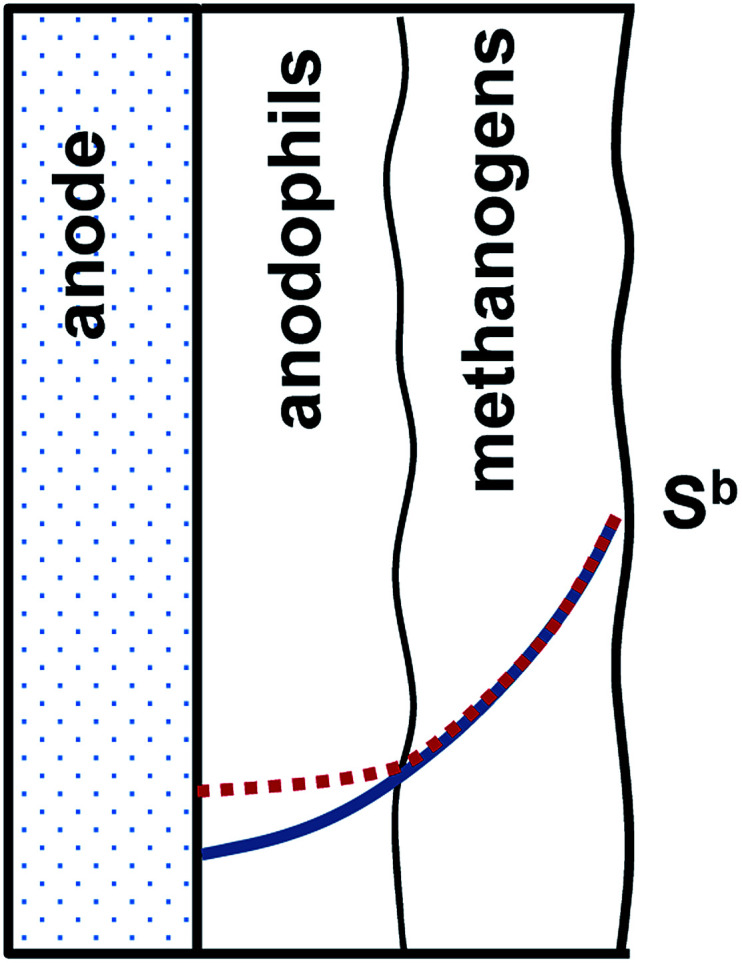
Conceptual model of carbon source distribution in a two layer electroactive anodic biofilm with anodophilic and methanogenic microbial populations. *S*^b^ is the bulk substrate concentration. Carbon source distribution in the biofilm corresponding to *U*_s_ > 0 (positive applied voltage) and *U*_s_ = 0 (no voltage) is shown by solid and dotted lines, accordingly.

To discuss implications of the on/off mode of MEC operation, a conceptual biofilm model can be proposed. The conceptual model shown in Fig. 6 considers electroactive and methanogenic microbial populations. While these populations can be evenly distributed in the biofilm, it is more likely to expect biofilm stratification, with the electroactive species predominantly present in the electrode (*e.g.* anode) vicinity. A similar species distribution might be expected at the cathode. Although both direct and mediator-based electron transfer mechanisms have been demonstrated in anodophilic species, the mechanism of direct electron transfer has the advantage of achieving a lower internal resistance, thus favoring anodophilic growth in close proximity to the electrode. Methanogenic microorganisms can be expected to populate the remaining biofilm. Moreover, an even more complex biofilm structure can be expected in a MEC fed with a more complex carbon source, *i.e.* fermentative bacteria are expected to proliferate at the biofilm surface.^27^

The proposed two-layer structure of the electroactive biofilm also agrees with the known kinetics of carbon source consumption by the anodophilic and methanogenic species. As compared to anodophilic bacteria, acetoclastic methanogens have lower specific growth rate and lower affinity to acetate, *i.e.* these species require a relatively high carbon source (acetate) concentration for growth.^22^ In a two-layer biofilm shown in Fig. 6, a gradient of carbon source within the biofilm is expected due to a combination of diffusion-limited carbon source transport and carbon source consumption, as discussed in several previous works.^27–29^ The two-layer biofilm structure enables both microbial populations to coexist, with methanogenic microorganisms exposed to higher acetate concentrations and anodophilic microorganisms growing at lower levels of acetate in the biofilm core. In fact, such two-layer structure could explain higher COD removal rates experimentally observed in MECs as compared to conventional anaerobic reactors.^30^

While periodic disconnection of the power supply (zero applied voltage) is not expected to directly affect metabolism of methanogenic populations, it prevents carbon source consumption in the anodophilic part of the biofilm at zero applied voltage corresponding to the “off” part of each cycle. High current observed immediately after the power supply is reconnected can be explained both by internal (double layer) capacitance of the biofilm and by higher carbon source concentration (availability) immediately after the power supply is reconnected. Considering higher growth rates of the anodophilic microorganisms as compared to the methanogens, it can be hypothesized that the on/off mode of operation provided long-term advantages and increased the fraction of electroactive anodophilic species within the biofilm. Confirmation of this hypothesis could be achieved by direct analysis of anodophilic and methanogenic microorganisms using biomolecular tools as well as by computer simulations using a distributed parameter biofilm model.

## 4. Conclusion

This study proposes a novel approach of MEC operation in which the power supply (applied voltage) is periodically disconnected. Although the on/off mode of operation does not appear to increase the average MEC current as compared to operation at a fixed applied voltage, it was observed to substantially improve long-term MEC performance. Estimations of MEC internal resistance before and after the on/off operation confirmed significantly lower internal resistance after prolonged on/off operation. Furthermore, both COD removal efficiency and the average current were observed to increase after the on/off operation. Although the proposed approach for MEC operation requires a more comprehensive study in order to clarify the underlying changes in microbial populations and to optimize duty cycle based on long-term MEC operation, the approach can be already considered for practical applications, where performance stability and high COD removal are of importance.

## Conflicts of interest

There are no conflicts to declare.

## Supplementary Material
